# Vehicle Scheduling Schemes for Commercial and Emergency Logistics Integration

**DOI:** 10.1371/journal.pone.0082866

**Published:** 2013-12-31

**Authors:** Xiaohui Li, Qingmei Tan

**Affiliations:** 1 College of Economics and Management, Nanjing University of Aeronautics and Astronautics, Nanjing, Jiangsu, China; 2 College of Foreign Languages, Yantai University, Yantai, Shandong, China; Rutgers University, United States of America

## Abstract

In modern logistics operations, large-scale logistics companies, besides active participation in profit-seeking commercial business, also play an essential role during an emergency relief process by dispatching urgently-required materials to disaster-affected areas. Therefore, an issue has been widely addressed by logistics practitioners and caught researchers' more attention as to how the logistics companies achieve maximum commercial profit on condition that emergency tasks are effectively and performed satisfactorily. In this paper, two vehicle scheduling models are proposed to solve the problem. One is a prediction-related scheme, which predicts the amounts of disaster-relief materials and commercial business and then accepts the business that will generate maximum profits; the other is a priority-directed scheme, which, firstly groups commercial and emergency business according to priority grades and then schedules both types of business jointly and simultaneously by arriving at the maximum priority in total. Moreover, computer-based simulations are carried out to evaluate the performance of these two models by comparing them with two traditional disaster-relief tactics in China. The results testify the feasibility and effectiveness of the proposed models.

## Introduction

The increasing complexity and magnitude of global emergency relief operations create a critical need for effective and efficient disaster-relief logistics. Disaster prevention, protection, and reconstruction are the major areas of focus to reduce human suffering and damage from disasters [1]. Quick response to the urgent relief needs right after disasters through efficient logistics dispatch is vital to the alleviation of disaster impact in the affected areas, which remains challenging in the field of logistics and related study areas.

The significance of issues on relief salvaging to areas suffering from disasters, e.g., drought and earthquakes, and the resulting logistics problems had been addressed previously in [2]–[11]. As for relief resource's delivery, transportation researchers have tackled different topics such as shortest path selection and vehicles schedule [12]–[22]. This range of studies has developed very consistent knowledge on how emergencies can be better managed throughout mitigation, preparedness, response and recovery.

Once an unexpected disaster strikes, quick salvaging efforts are immediately made by governmental agents, non-profit organizations or individual volunteers. Moreover, at the same time, upon the government's instruction, some commercial logistics companies immediately participate in the relief operation by proving efficient and effective transportation service. As argued by Lyles [23], there is always a fierce need for coordinating the logistics resources of public and private sectors to avoid arbitrary resource allocation during disasters. In practice, compared with non-professional organizations, the private-owned logistics companies have turned out to be the backbone in disaster-relief activities. That is, besides the ordinary inter-regional commodity exchange, commercial logistics companies also play an important role in emergency relief distribution. Thus, there emerges a practical concern for a commercial logistics company as to how to coordinate the commercial and emergency logistics operation with the purpose of performing relief dispatch obligation with as little as a possible negative impact on commercial transport safety.

Some specific features of the emergency transportation problems were discussed in previous literatures. However, the commercial-and-emergency logistics integration problem received far less attention. One of the reasons lies in the differences between commercial logistics and emergency logistics. Commercial logistics has been clearly defined in the previous literature [24]–[26]as “Logistics is the process of planning, implementing, and controlling the efficient, effective flow and storage of goods, services and related information from the point of origin to the point of consumption for the purpose of conforming to customers' requirements at the lowest total cost”. But the definition of emergency logistics has not yet been well clarified. Another reason is illustrated by Wei Yi and Arun Kumar who hold that emergency logistics support and vehicle dispatch have features different from the established commercial dispatch settings [27].

Despite these differences, the commercial and emergency logistics systems share some common grounds in many aspects, which have formed a concrete foundation for their integration. First of all, both consider material items, number of vehicles, modes of transportation, number of depots, demand of materials, transportation networks, vehicle capacity, travel time on the route, and various operational modes. Their objectives are to seek for a combination of those variables that minimize total traveling time, minimize size of the vehicle fleet, maximize service capacity, and minimize fixed and variable costs [28]. The minor difference between these two systems lies in that in addition to efficiency objective, emergency logistics also pay emphasis on fairness. Secondly, similar to commercial distribution systems, emergency distribution systems also consist of three separate parts: demand, supply, and transportation. The collection points of commodities in non-devastated areas play the role of supply, while the demand points are the devastated areas where relief resources are provided to victims who play the role of customers. The only difference is that the distribution depots are temporary storage points instead of a permanent distribution warehouse.

In China, two traditional scheduling schemes are prevailing for emergency logistics schedule. For the first scheme, denoted by scheme 1, the available vehicles are utilized in emergency logistics business with no commercial business involved. The logistics company, therefore, gains profits of governmental subsidies minus relevant costs, and at the same time suffers losses occurring from idle vehicles. For the second scheme, denoted by scheme 2, the logistics company can accept commercial business on condition that the disaster-relief requirements are satisfied and there is not any commercial business to be performed on hand. Thus, the daily earnings for the company are emergency logistics subsidies plus commercial profits.

In this study, we propose two vehicle scheduling schemes using linear multi-objective programming approaches to obtain the maximum commercial profits while satisfying the disaster-relief requirements. The first one, denoted by scheme 3, is a prediction-based model. It schedules vehicles according to the predicated emergency and commercial business amount to ensure the maximum commercial profits. The other one, denoted by scheme 4, is associated with priorities of emergency and commercial logistics business, and it is capable of satisfying their joint requirements by achieving the total maximum priorities. Both proposed models feature such objectives as minimizing the total cost, maximizing the minimal satisfaction, and maximizing commercial profits in the scheduling process. Furthermore, to verify their effectiveness and feasibility, we compare them with other two traditionally utilized models. The simulation results present the advantages of these two newly-proposed schemes.

The remainder of this paper is organized as follows. In Section 2, assumptions and constraints are described to explain the models in the successive sections. Traditional and new scheduling schemes are discussed in Section3. In Section 4, simulations are made to demonstrate the feasibility and effectiveness of these models. Conclusions and recommendation for future research are summarized in Section 5.

## Assumptions and Constraints

### Assumptions

1. Resources possessed by a logistics company, including vehicles, warehouses, drivers, do not change during the disaster-relief period.
2. The accessorial costs related to goods collection in source areas, vehicle maintenance, the staffs' salaries, and the like, are not considered.
3. As for commercial logistics, any transportation requirement is conducted between the headquarters (hereinafter called the source point) and its branch companies (hereinafter called the transfer depots). The transfer depots involved may not interact with one another.
4. Disaster-relief activity is limited to a certain period, during which any emergency distribution task is fulfilled between the source point and demand points. Besides, vehicles are assumed to be loaded with relief materials merely when leaving for destination.
5. Required amount for emergency logistics tasks is within the scope of a company's ability.
6. The governmental subsidies for relief distribution are less than profits earned by the logistics companies from participation in commercial logistics business.
7. The operation cost and wear/tear cost are paid in the form of salaries, and these costs are insignificant compared to the fuel consumption in vehicle logistics, and thus these costs can be neglected.

### Symbol explanation

The definitions of parameters and variables specified for this study are summarized as follows.

**Table:** 

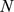	total sum of transfer depots
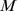	number of vehicles
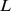	item number of commercial logistics
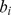	specified loading weight of the 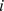 -th vehicle, i = 1,2, 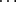 ,M
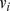	specified loading volume of the 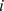 -th vehicle, i = 1,2, 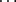 ,M
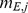	emergency-logistics business' weight of the 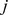 -th item, j = 1,2, 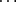 ,L
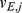	emergency-logistics business' volume of the 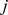 -th item, j = 1,2, 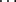 ,L
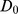	distance between the headquarters and relief demand points
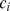	fuel consumption per ton and per kilometers for the 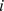 -th vehicle, i = 1,2, 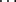 ,M
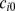	fuel consumption per kilometers for the 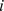 -th unloading vehicle, i = 1,2, 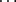 ,M
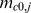	total weight of commercial business carried from the headquarters to the 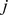 -th
	transfer depot, j = 1,2, 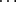 ,N
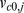	total volume of commercial business carried from the headquarters to the 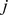 -th
	transfer depot, j = 1,2, 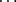 ,N
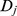	distance between the headquarters and the 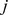 -th transfer depot, j = 1,2, 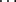 ,N
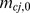	total weight of commercial business loaded from the 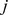 -th transfer depot to the
	headquarters, j = 1,2, 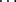 ,N
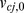	total volume of commercial business loaded from the 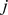 -th transfer depot to the
	headquarters, j = 1,2, 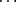 ,N
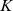	total amount of emergency logistics business
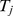	the 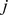 -th emergency vehicle state. If it is used, 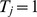 , otherwise 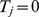
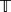	set of emergency vehicle states
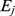	set of business fulfilled by the 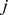 -th emergency vehicle
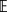	set of business fulfilled by all emergency vehicles
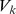	the 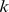 -th commercial vehicle state. If it is used, 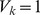 , otherwise 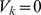
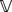	set of commercial vehicle states
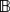	set of all transfer depots
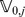	set of vehicles departing from the source point to the 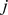 -th transfer depot
	j = 1,2, 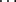 ,N

### Charging principles

For a commercial logistics business, the transportation charges are collected in accordance to the principle
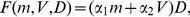
(1)where 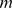 is the weight of commodities, 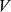 is the volume of commodities, 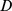 is the distance from the source point to the demand point, and 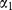, 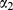 are the coefficients for each unit of weight and volume per kilometer, respectively. However, for emergency logistics business, the governmental subsidies are paid following the rule of
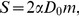
(2)where 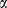 is the subsidies for one unit of weight per kilometer, and 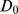 is the distance between the source point and the affected point. In theory, 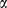 is less than 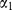 in (1).

## Methods

### Emergency-logistics-based scheduling model

In this model, the disaster-relief materials are dispatched as many as a logistics company can. The income to the logistics company obtained is the difference between the government's subsidies and the cost incurred from delivering relief commodities. The profits equation may be formulated as
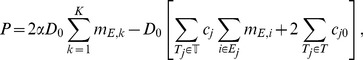
(3)and thus the scheduling model is
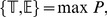
(4)s.t.
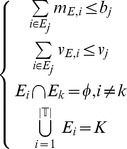



### Commercial-logistics-based Scheduling Model

In this model, the profit margin is the difference between the income paid by customers and the costs incurred as mentioned above. Suppose the set of vehicles engaged in commercial logistics is 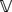, then the optimal scheduling model for commercial logistics business is
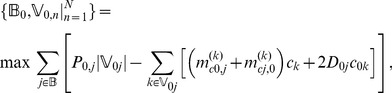
(5)s.t.
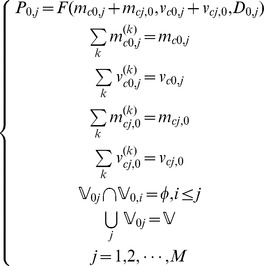



Under the disaster-relief circumstances, the daily economic loss for a logistics company is equal to the commercial profit obtained through this scheduling process when idle vehicles exist.

### Prediction-based Scheduling Scheme

The main objective of this scheme is to maximize commercial profit based upon the prediction of emergency and commercial business in advance. Therefore, the logistics company is able to assign emergency transportation tasks, and at the same time accept orders from commercial organizations. Because the commercial logistics transportation modeling may be understood as a steady process, p-order auto-regressive (AR) model can be used to predict its amount. Besides, in view of time-varying characteristic of emergency logistics business, a simple gradient method is adopted rather than AR model. Let the commercial business amount accepted on the 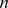-th day be 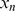, then the AR(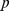) modeled is given by
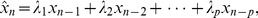
(6)where 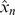 is the predicted business amount in the 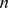-th day, 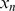 is the real business amount, and 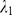, 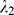, , 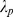 are the weight coefficients.

If the prediction error is 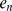, i.e. 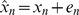, then 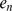 is minimized when it is not related with the measured value, that is
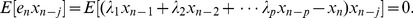
(7)


Suppose 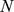 is the acquired data length, then the autocorrelation function can be written as
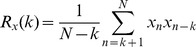
(8)
Equation (7) can be expanded as
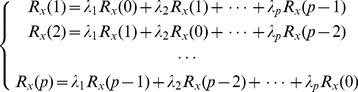
(9)


The values 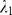, 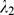, , 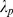 can be obtained by solving the above equations. The estimation variance of the AR(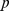) model is
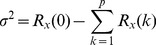
(10)


As for emergency logistics, estimates of possible demand over specific periods of time are made based on emergency-coordination capability, the extent of devastation, and the age and sex combination of victims at the relief-demand points who need care [28]. In arriving at a more reasonable prediction of emergency business volume 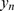, the gradient prediction method is applied in our research. In emergency context, the demand for relief materials is extremely heavy in the initial stage after a disaster strikes, whereas with the disaster situation leveling off, the urgent demand grows stable. That is, the emergency demand may be reckoned as a gradually changed value, which conforms to the conditions of gradient prediction. The prediction formula is expressed as
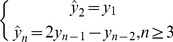
(11)


The scheduling method is made up of two steps. In the first step, 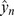 is estimated, and then based on the estimate, the vehicles are scheduled and the commercial vehicles set is figured out. In the second step, 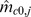, 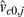, 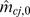 and 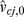 are estimated, and then an optimal scheduling scheme based on (5) is worked out by pointing out an acceptable amount of commercial business.

The actual profits in this scheme are emergency logistics subsidies plus commercial logistics business profit. If no idle vehicle exists, no losses will occur; if the predicted business volume is greater than the actual one, there will be idle vehicles and the losses thus incurred are equal to the highest-profit business sacrificed.

### Priority-based Scheduling Scheme

**Figure 1:**
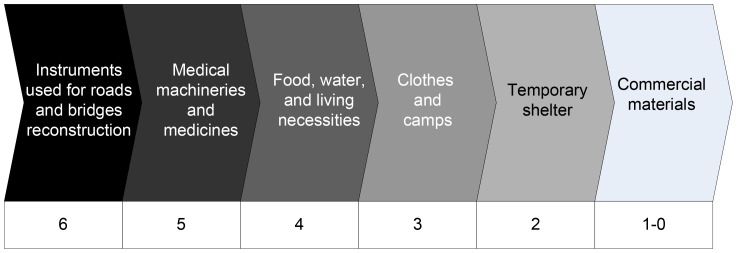
Business priority diagram.

The priorities of a logistics business are designed as preliminary preparation for logistics transportation scheduling (shown in Figure 1) in accordance with the properties of the cargoes to be delivered. The priorities of commercial logistics business, ranging from 0 to 1, are given by 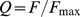,where 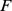 is the expenses paid for this business, and 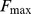 is the maximum expenses among the whole lot of commercial business. In addition, the emergency logistics business ranks from 2 to 6.

The scheduling process begins by determining the business according to current business amount and its priority. The mathematic model is described as follows.
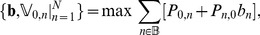
(12)s.t.
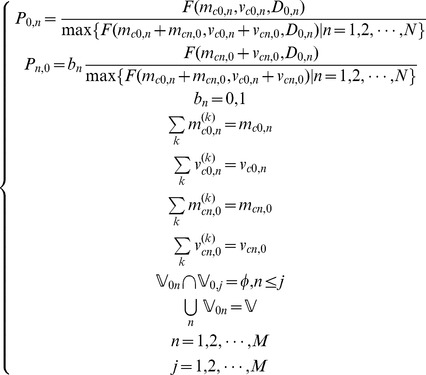



In this model, 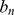 is a binary variable used to denote the destination. If 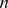 is the label of a disaster-relief area, then 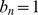, otherwise 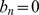.

The vehicles will depart from the source point as soon as they are fully loaded. If a business belonging to higher priority arrives but no extra vehicle is available, or if the vehicles are unable to fulfill all high-priority business due to insufficient loading capacity, the on-going low-priority business will pause, and the associated vehicles will be required to unload resources at the nearest transfer depot and then carry another lot of cargoes back to the source point to perform the high-priority business. The vehicles in question will not continue their original commercial business until the specific emergency transportation task is finished.

This scheme integrates commercial business and emergency business together based on priority determination. However, it generates a need for more wear/tear operations and also increases storage pressure. The solution is to control the amount of commercial business in a reasonable range by predicting emergency logistics business in advance. For instance, when the vehicles are left less after emergency business is assigned, only the commercial business whose destination is directly connected to the source point can be accepted. If the vehicles left are in a large quantity, long-distance commercial business can be accepted. Note that the transshipment charges may be omitted with respect to the large overall logistics profits.

## Results and Discussion

**Figure 2:**
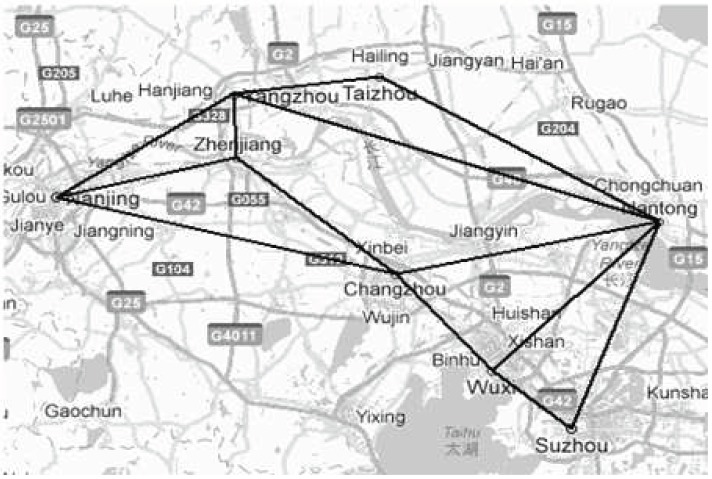
Transportation network of Southern Jiangsu Province.

To evaluate the performance of the two newly-proposed scheduling models mentioned above, we compared them with the traditional disaster-relief methods in China by using computer-based simulations. The simulations take Southern Jiangsu Province of China as an instance, and the transportation network is shown in Figure 2.

### Simulation scenario

Assume that a logistics company locates its headquarters in Nanjing, the provincial capital of Jiangsu province, and its seven branch companies in Yangzhou, Zhenjiang, Changzhou, Wuxi, Suzhou, Taizhou, and Nantong. The distances between Nanjing, the source point, and its branches, the transfer depots are listed in Table 1. Meanwhile, the logistics company has three types of vehicles (see Table 2). Table 3 presents the average commercial business amount.

**Table 1:** The distances from Nanjing to its branches (km).

	Yangzhou	Zhenjiang	Changzhou	Wuxi	Suzhou	Taizhou	Nantong
Nanjing	100	80	136	178	214	168	265

**Table 2:** Details of vehicle parameters.

Types	Number	Loading-weight	Loading-volume	Transport Cost	Non-loading cost
	(set)	(ton)	( 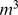 )	(Yuan/ton  km)	(Yuan/km)
Overlarge-sized	4	20	20	0.75	0.5
Large-sized	4	16	16	0.7	0.45
Middle-sized	2	10	10	0.6	0.4

**Table 3:** Average commercial business amount between Nanjing and other transfer depots.

From: Nanjing		To	From	To: Nanjing	
Weight(ton)	Volume( 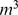 )			Weight(ton)	Volume( 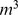 )
15	8	Yangzhou		20	20
12	5	Zhenjiang		15	15
25	15	Changzhou		25	16
25	10	Wuxi		20	10
15	10	Suzhou		15	26
25	20	Taizhou		30	25
20	15	Nantong		20	25

**Figure 3:**
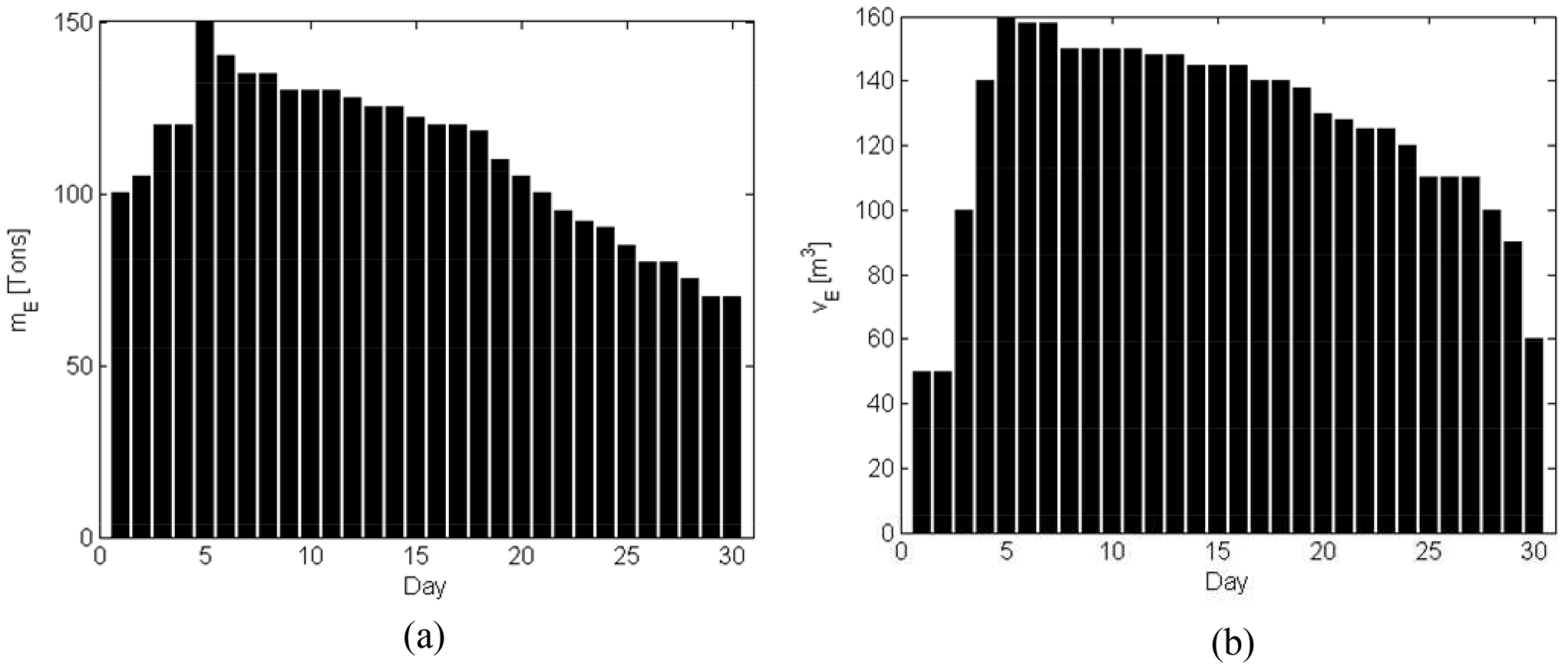
Disaster-relief materials delivery assigned to the logistics company. (a) Weight. (b) Volume.

Suppose Nantong is the disaster-affected area, the relief transport tasks assigned by the government are shown in Figure 3.

**Figure 4:**
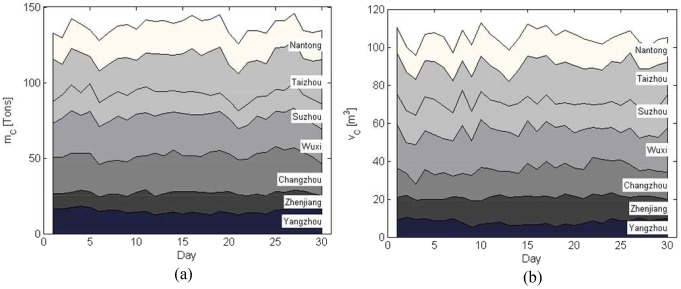
Commercial business amount in Nanjing headquarters. (a) Weight. (b) Volume.

During the disaster-relief process, the logistics company may accept some commercial business at the same time. Figure 4 illustrates the business variations in terms of the commercial business amount to be delivered to Nanjing headquarters.

**Figure 5:**
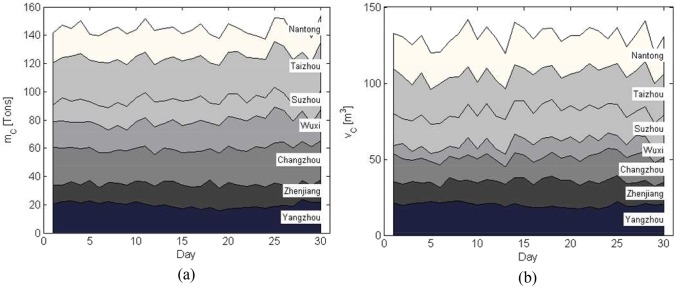
Commercial business amount in other depots. (a) Weight. (b) Volume.

Similarly, commercial business is also conducted from each transfer depot to the headquarters continuously. The business amounts are shown in Figure 5 with respect to weight and volume, respectively.

### Simulation Experiment and Discussion

**Figure 6:**
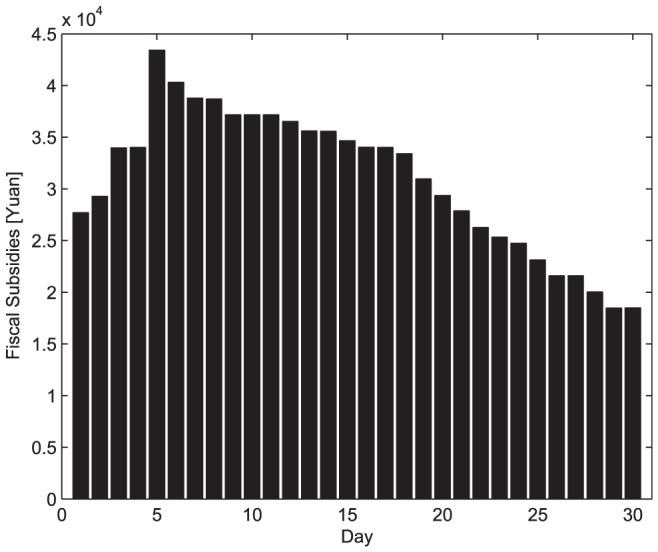
The profits of emergency logistics.

**Figure 7:**
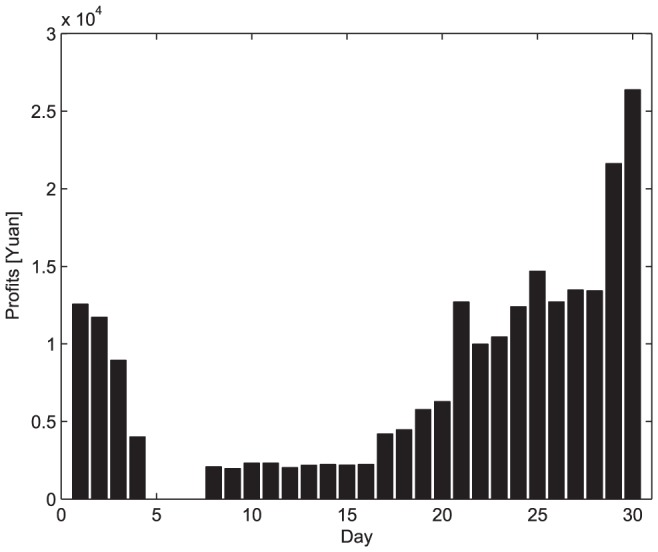
The optimal commercial profits.

In any case, emergency requirements must be given higher priority and be satisfied first of all. According to (3), the profits of the emergency logistics can be calculated (shown in Figure 6). According to (4), the optimal commercial profit is obtained by using the remainder vehicles on condition that the emergency logistics are fulfilled. From Figure 3 and Figure 6, it can be seen that the emergency profits are in line with the disaster-relief requirements. Figure 7 shows that with the demand reduction of the disaster-relief, more vehicles can be scheduled to perform the commercial logistics and thus the commercial profit increases.

The two traditional schemes may achieve the objectives of the commercial-and-emergency logistics framework in some sense. The new schemes, however, are aimed to optimize the dispatch scheduling operation based on demand prediction and priority consideration, respectively.

**Figure 8:**
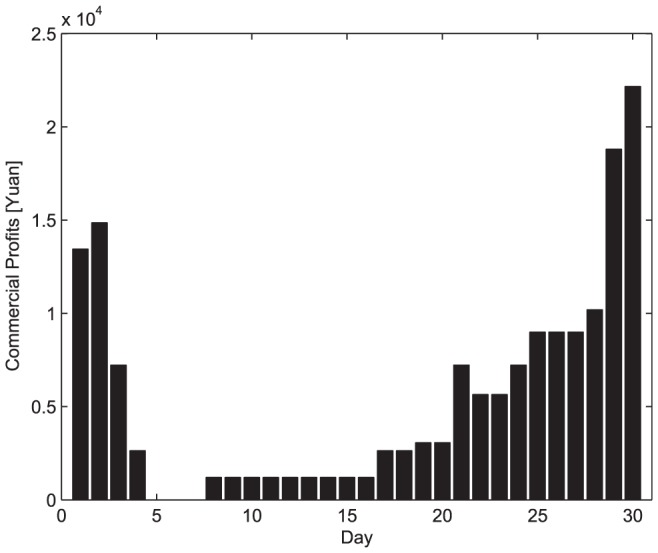
The commercial profits in scheme 2.

As for scheme 1, it can not provide commercial profits because all the vehicles are ready to fulfill the disaster-relief requirements. As for scheme 2, the commercial logistics profits are shown in Figure 8. However, it allows the logistics company to accept new commercial businesses until the existing commercial businesses are completed, the coming commercial businesses that can provide the maximum profits will be refused, and thus this scheme will not provide an optimal resolution.

**Figure 9:**
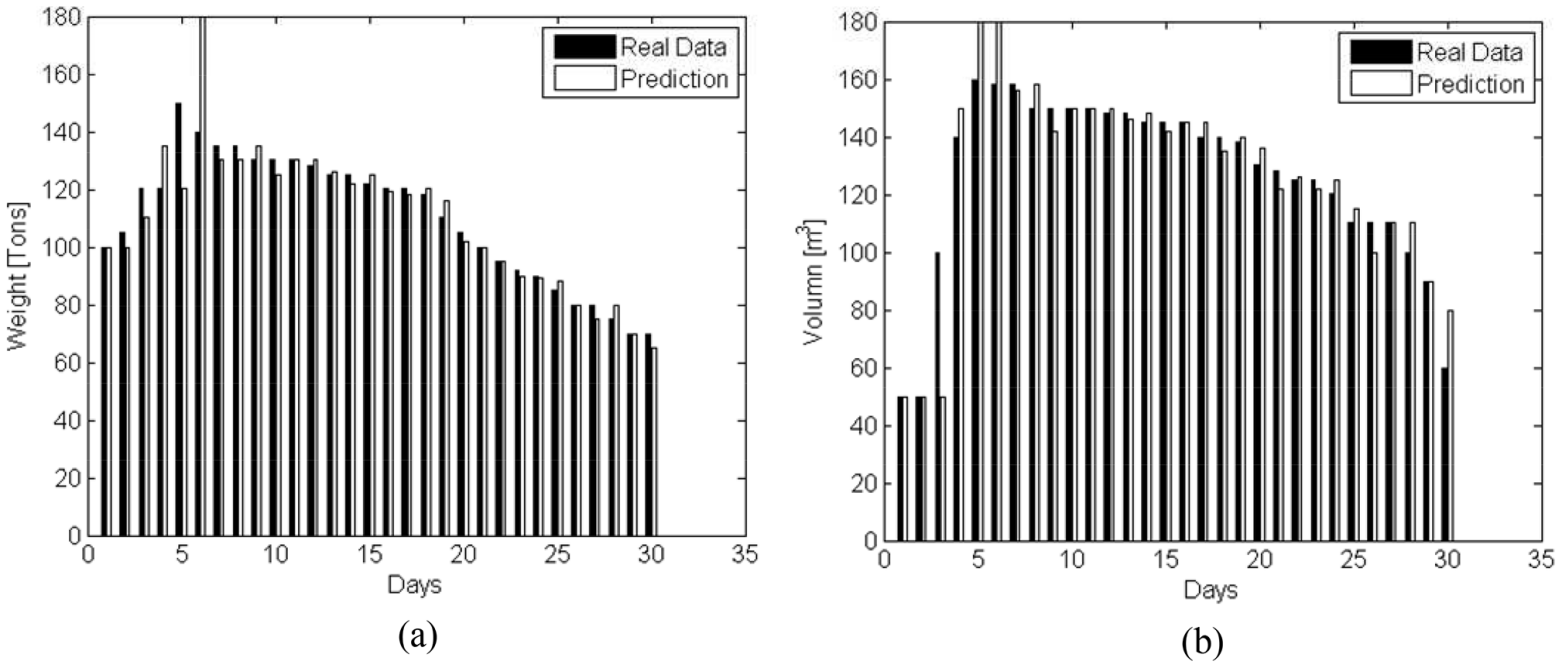
Real and predicted amounts of the disaster-relief materials. (a) Weight. (b) Volume.

**Figure 10:**
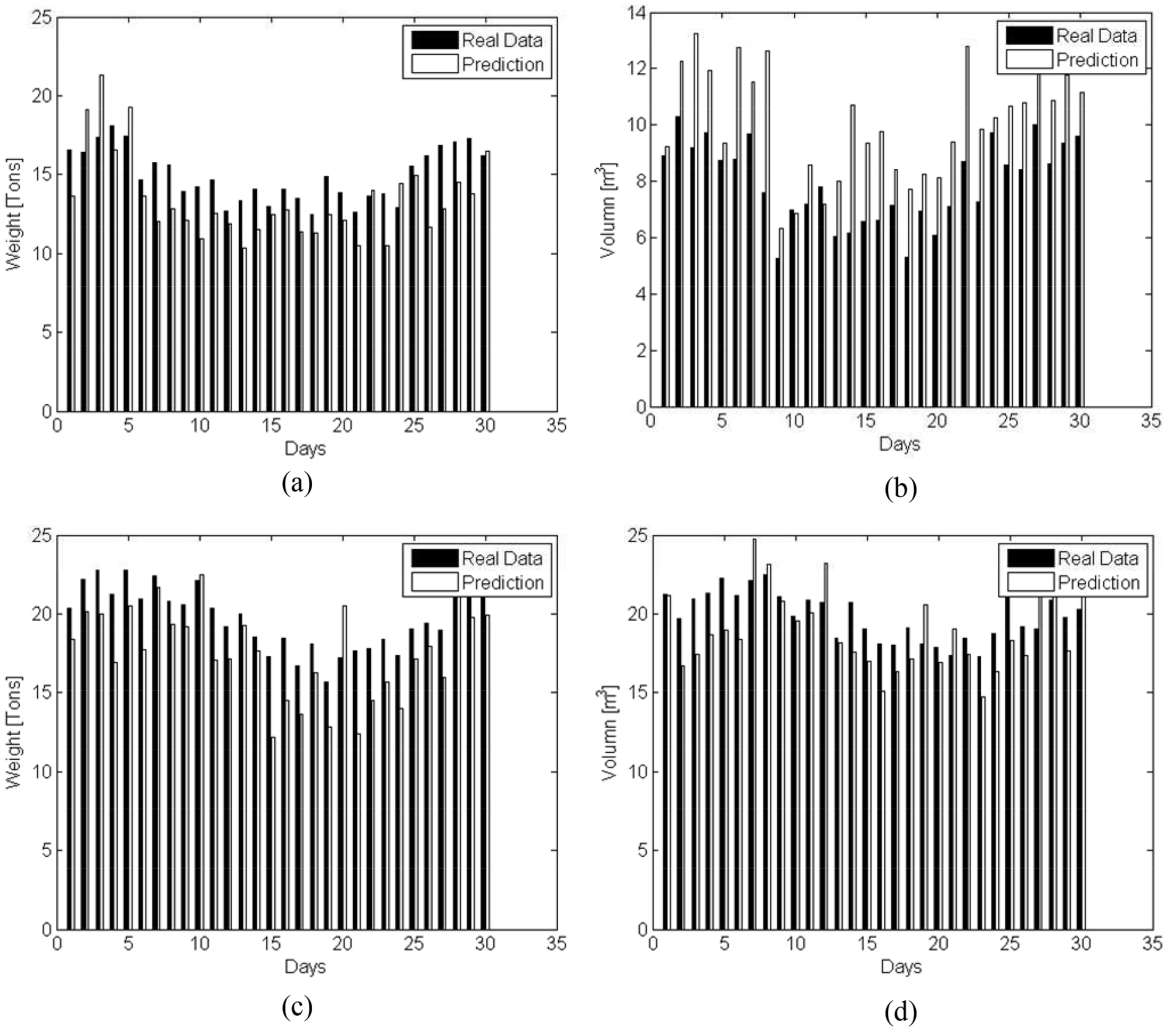
Real and predicted results of commercial logistics. (a) Weight(From Nanjing to Yangzhou). (b) Volume(From Nanjing to Yangzhou). (c) Weight(From Yangzhou to Nanjing). (d) Volume(From Yangzhou to Nanjing).

As for scheme 3, the predicted emergency business amounts are presented in Figure 9, where the filled bars indicate real data, and the transparent ones denote the predicted value. For simplification, we only present the predicated results of the commercial business amount from Nanjing to Yangzhou, and that from Yangzhou to Nanjing in Figure 10.

**Figure 11:**
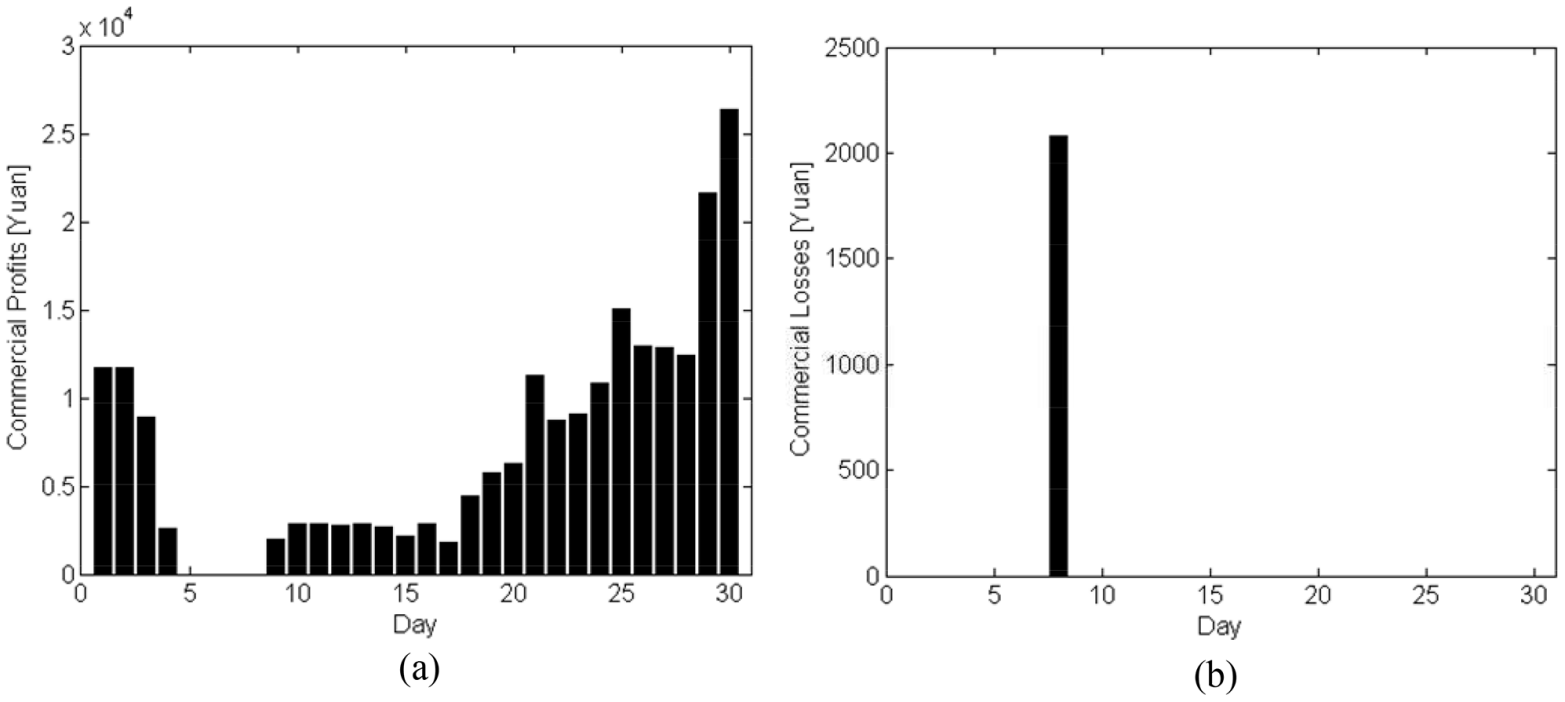
The scheduling results of scheme 3. (a) Commercial Profits. (b) Commercial Losses.

The commercial profits provided by scheme 3 are shown in Figure 11(a). Figure 11(a) shows a similar intent with Figure 8 except that the amplitude of Figure 11(a) is larger. It lies in that the scheme 3 can predict the incoming commercial business and gives a reasonable scheduling of the commercial requirements. However, the estimation error may lead to commercial losses. Lower diaster-relief requirement estimate may cause the accepted commercial business to be stranded and thus the company should compensate the customer for the loss. Higher diaster-relief requirement estimate may result in idle vehicles, which leads to the commercial business loss. Figure 11(b) shows a circumstance of overestimation, where commercial loss appears due to the idle vehicles.

**Figure 12:**
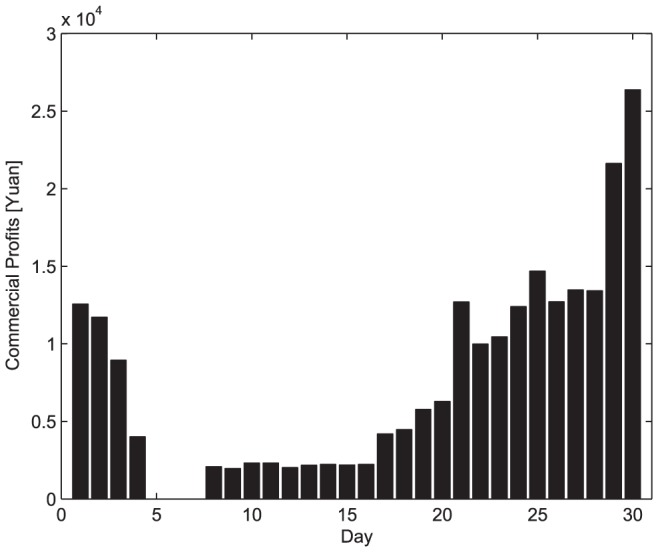
The commercial logistics profits in Scheme 4.

In scheme 4, the commercial profits are obtained as shown in Figure 12. Because the scheme schedules the vehicles for both emergency and commercial logistics in a unified form, no idle vehicles appear, and an optimal solution can be obtained.

To compare the performance of the four schemes, the individual commercial profits are summarized by Table 4. From this table and Figure 7, Figure 8, Figure 11, and Figure 12, we can easily draw conclusions as follows.

**Table 4:** Commercial profits provided by the four schemes.

Scheme	Scheme 1	Scheme 2	Scheme 3	Scheme 4
Commercial profits (Yuan)	0	164220	215340	225250

1. For scheme 1, there are not any commercial profits since all vehicles are reserved for emergency logistics, that is, the commercial losses are related with the idle vehicles;
2. For scheme 2, although the logistics company accepts some commercial business, the profits are at a lower level because it fails to make optimal scheduling in dispatch operation. Obviously, the two traditional dispatch schedules are not capable of achieving optimal efficiency;
3. Scheme 3 and 4 improves greatly in protecting the logistics company's commercial benefits. In more detail, Scheme 3 brings about higher commercial profit via preliminary emergency dispatch predication. However, owing to prediction error, commercial losses may occur accordingly. Scheme 4 may be a better dispatch scheduling approach in that its commercial profit is almost as same as that shown in Figure 7.

## Conclusions

Two vehicle scheduling models are proposed in this research in order to bring about maximum commercial profits for a logistics company while it is carrying out government-assigned emergency logistics. One is based on the predication method, and the other is based on business priority-giving. The simulation results show that the two new schemes can provide better performance than the traditional scheduling schemes in China.

The predication-based scheme may result in high profits because it can predicate both the emergency and commercial business and thus it can schedule the vehicles based on the estimates to determine the commercial requirements that can provides maximum profits. So in reality, when other kinds of commercial businesses arrive, the company can refuse them. However, idle vehicles and overstock problem may appear owing to predication error. The priority-based scheme may achieve higher profits because the vehicles are scheduled and utilized more efficiently.

In the proposed models, we assume that the transfer depots do not interact with one another. However, to meet irregular requirements in emergency condition, all the transfer depots may cooperate with one another to carry out the commercial business that are probably refused by other logistics companies. Furthermore, cargo grooming schemes should also be considered under the emergency condition. We will focus on these problems in the next phase.
